# Feasibility and Impact of Remote Glucose Monitoring Among Patients With Newly Diagnosed Type 1 Diabetes: Single-Center Pilot Study

**DOI:** 10.2196/33639

**Published:** 2022-01-17

**Authors:** Stephanie Crossen, Crystal Romero, Allison Reggiardo, Jimi Michel, Nicole Glaser

**Affiliations:** 1 Department of Pediatrics University of California, Davis Sacramento, CA United States; 2 Center for Health and Technology University of California, Davis Sacramento, CA United States

**Keywords:** remote monitoring, type 1 diabetes, pediatrics, diabetes, T1D, patient-generated data, mobile health, application

## Abstract

**Background:**

Caregivers of children with newly diagnosed type 1 diabetes (T1D) maintain close contact with providers for several weeks to facilitate rapid adjustments in insulin dosing regimens. Traditionally, patient glucose values are relayed by telephone for provider feedback, but digital health technology can now enable the remote sharing of glucose data via mobile apps.

**Objective:**

The aim of this study was to test the feasibility of remote glucose monitoring in a population of children and adolescents with newly diagnosed T1D and to explore whether remote monitoring alters habits for self-review of glucose data or perceived ease of provider contact in this population as compared to a nonrandomized control group.

**Methods:**

Data were collected from families who chose to participate in remote monitoring (intervention group) as well as from patients receiving usual care (control group). The intervention group received Bluetooth-capable glucose meters and Apple iPod Touch devices. Patient-generated glucose data were passively relayed from the meter to the iPod Touch and then to both the electronic health record (EHR) and a third-party diabetes data platform, Tidepool. The principal investigator reviewed glucose data daily in the EHR and Tidepool and contacted the participants as needed for insulin dose adjustments during the time between hospital discharge and first clinic appointment. Families in the control group received usual care, which involved keeping written records of glucose values and contacting the diabetes team daily by telephone to relay data and receive treatment recommendations. A total of 40 families (20 for the intervention group and 20 for the control group) participated in the study. All families were surveyed at 1 month and 6 months regarding self-review of glucose data and ease of contacting the diabetes team.

**Results:**

Patient-generated glucose data were remotely accessible for 100% of the participants via Tidepool and for 85% via the EHR. Survey data indicated that families in the intervention group were more likely than those in the control group to review their glucose data using mobile health apps after 1 month (*P*<.001), but by 6 months, this difference had disappeared. Perceived ease of contacting the clinical team for assistance was lower for the intervention group after 6 months (when receiving usual care) in comparison to during the intervention period (*P*=.48) and compared with a control group who did not have exposure to remote monitoring (*P*=.03).

**Conclusions:**

Remote glucose monitoring is feasible among pediatric patients with newly diagnosed T1D and may be associated with the earlier adoption of mobile health apps for self-management. The use of broadscale remote monitoring for T1D in the future will depend on improved access to Bluetooth-enabled mobile devices for all patients, improved interoperability of mobile health apps to enable data transfer on Android as well as Apple devices, and new provider workflows to handle large-scale panel management based on patient-generated health data.

**Trial Registration:**

ClinicalTrials.gov NCT04106440; https://clinicaltrials.gov/ct2/show/NCT04106440

## Introduction

The management of type 1 diabetes (T1D) is labor-intensive and data-driven for both patients and providers. The advent of continuous glucose monitoring (CGM) devices and insulin pumps has dramatically increased the volume of patient-generated health data (PGHD) available for T1D management [1,2], and mobile health apps have begun to make these data accessible remotely. However, advanced therapeutic technologies such as pumps and CGM are less frequently utilized by racial or ethnic minority patients and those from low-income households [3], and they are often not available directly after diagnosis due to payor restrictions. In addition, data from most insulin pumps can only be shared remotely using broadband internet, making this type of remote monitoring less feasible for individuals from racial or ethnic minorities or low-income households, who are more likely to depend on cellular devices for internet access [4]. Remote access to intermittent self-monitoring of blood glucose (SMBG) data has also become possible in the last five years via Bluetooth-enabled glucose meters, over a dozen of which are now commercially available [5]. SMBG meters remain the standard of care for T1D [6] and are provided to patients at the time of diagnosis; therefore, the remote monitoring of SMBG data via a mobile device has the potential to be broadly applicable within the T1D patient population.

The period directly after diagnosis of T1D involves frequent (often daily or biweekly) interaction between patients and providers to review glucose trends and adjust insulin doses to account for the effects of partial remission (“honeymoon phase”) and changes in diet and activity. These interactions have traditionally taken place via telephone but are well suited to remote monitoring. This pilot study evaluated the feasibility of remote glucose monitoring among pediatric patients with newly diagnosed T1D by using mobile devices and digital health apps to relay intermittent patient-generated glucose values into both the electronic health record (EHR) and a secure, third-party platform designed for diabetes data management [7]. This study also explored whether the use of remote monitoring in this patient group may alter habits for self-review of glucose data and the perceived ease of contacting providers for help during 6 months after diagnosis, as compared to a control group receiving usual care.

## Methods

### Setting

This study took place at the University of California, Davis (UCD) medical center in Sacramento, California. At our medical center, children with newly diagnosed T1D are typically hospitalized for 2-3 days for initiation of insulin therapy and to receive caregiver education about home T1D management. After hospital discharge, patients are scheduled for an appointment in the pediatric diabetes clinic approximately 2-6 weeks later, depending on availability. During the time between hospital discharge and first clinic appointment, caregivers are instructed to record patients’ glucose measurements on a daily basis and contact the clinic team or on-call pediatric endocrinologist by telephone to relay these glucose values and discuss any needed dose changes.

### Recruitment

In this nonrandomized study, the recruitment of participants for the intervention and control groups took place separately. The participants for the intervention group were recruited during their initial hospitalizations at the time of T1D diagnosis. Inclusion criteria for children in the intervention group were (1) aged 1-17 years, (2) newly diagnosed with T1D during current hospitalization, (3) daily access to the internet via Wi-Fi, and (4) planning to receive care from the UCD pediatric diabetes clinic for the next 6 months. The participants for the control group were recruited during their first visits to the UCD pediatric diabetes clinic. Inclusion criteria for children in the control group were (1) aged 1-17 years, (2) newly diagnosed with T1D 2-6 weeks earlier, (3) not already enrolled in the intervention arm of the study, and (4) planning to receive care from the UCD pediatric diabetes clinic for the next 6 months. The control participants included children who were diagnosed with T1D during the 6 weeks prior to study initiation, families whom the research team was unable to approach for the intervention arm at the time of diagnosis (due to limited staff availability), and 3 families who had declined the intervention due to a preference to receive usual care. The only exclusion criterion for participants in either group was if a primary caregiver did not speak English, due to concern that the mobile health apps did not offer non-English versions and could therefore introduce a communication barrier as compared to usual care. Twenty participants were recruited to each study arm.

The recruitment for this study took place between October 2019 and October 2020. All aspects of the study were reviewed and approved by the UCD Institutional Review Board and were conducted in accordance with COVID-19–related policies enacted by the UCD Office of Research. The study was also registered on ClinicalTrials.gov (NCT04106440).

### Intervention

**Figure 1:**
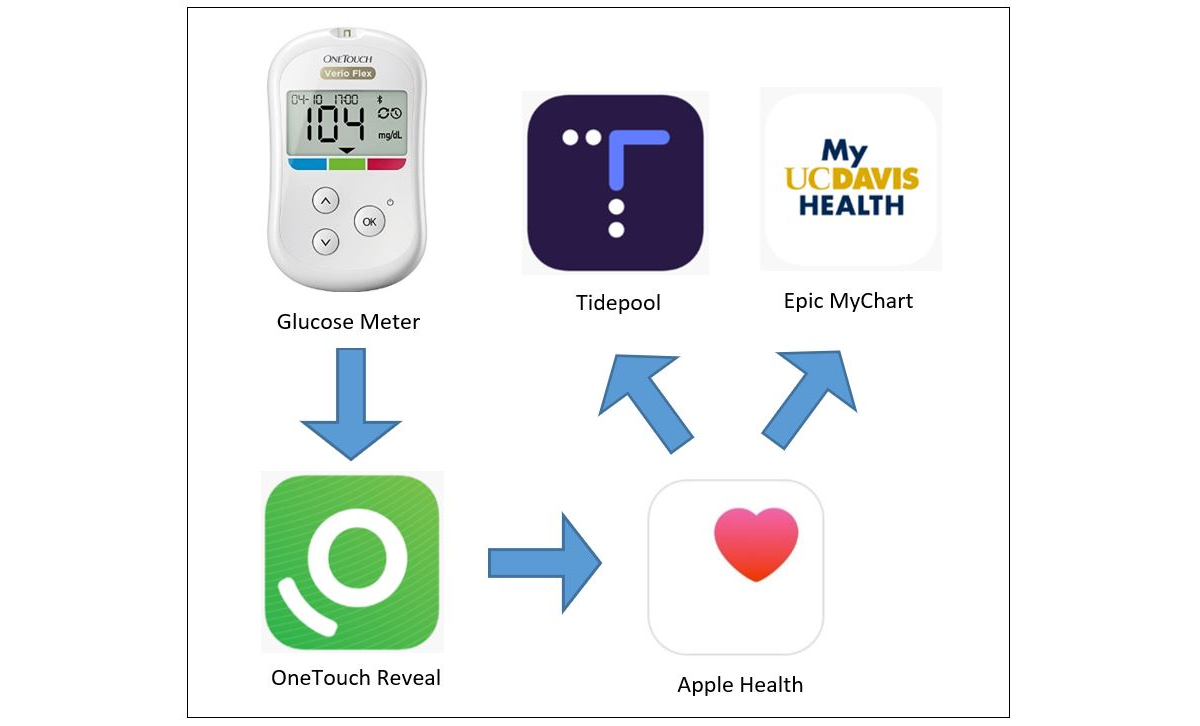
Relay of glucose data via mobile health apps.

The families in the intervention group were given a Bluetooth-capable glucose meter (OneTouch Verio Flex) and Apple iPod Touch device and instructed on their use prior to hospital discharge. Mobile health apps (OneTouch Reveal, Apple Health, Tidepool, and Epic MyChart) were installed on each iPod to facilitate the relay of patient-generated glucose data to provider-accessible platforms. As depicted in Figure 1, glucose data were transmitted via Bluetooth from the meter to the mobile device’s OneTouch Reveal app, then via Apple Health to Tidepool and Epic MyChart apps on the same device. When the device connected to Wi-Fi, these apps transmitted data to the cloud, making them viewable by the providers in Epic (via a flowsheet within the patient’s chart) and Tidepool (via the patient profile, which was linked to the clinic’s account). Research staff assisted the study participants in establishing user profiles within these apps and enabling data sharing between them.

After hospital discharge, the principal investigator reviewed glucose data for children in the intervention group daily in both Tidepool (a secure web-based platform for diabetes data management) [8] and Epic (the EHR used at UCD) and called their caregivers to discuss any needed changes in insulin dosing. This continued until each child’s first visit at the pediatric diabetes clinic. Children in the control group received usual care between hospitalization and first clinic visit, as described above under “Setting.”

### Data Collection

At the time of enrollment, demographic data, including age, sex, race, ethnicity, and insurance type, were abstracted from the EHR for each participant in order to characterize the study population. In addition, clinical data were abstracted from the EHR for all participants 6 months after diagnosis—including their most recent hemoglobin A_1c_ values, and whether they were using CGM and insulin pump technology for diabetes management—in order to identify any significant clinical differences between the study groups. At 1 month and 6 months after diagnosis, a brief survey of multiple choice questions was administered to the participants’ caregivers. This survey asked about typical frequency and modality for the self-review of glucose data and about the perceived ease of contacting the clinical diabetes team for help between clinic visits. For control participants who were enrolled >1 month after diagnosis, the 1-month survey was administered at the time of enrollment.

### Analysis

Demographic and clinical characteristics of the intervention and control groups were compared using the Fisher exact test for categorical variables and the Student *t* test for the means of continuous variables. The feasibility of remote glucose monitoring was assessed by the proportions of intervention participants for whom patient-generated glucose data were successfully relayed into (1) Tidepool and (2) the EHR for the duration of the intervention period. The differences in survey responses for the study groups at 1 month and 6 months were evaluated using the Fisher exact test.

## Results

### Study Population

The demographic characteristics of children in the control and intervention groups were similar at the time of enrollment except that the intervention group was significantly older, with a mean age of 11.3 years (SD 3.9) versus 8.4 years (SD 3.3) for the control group, *P*=.02 (Table 1).

The 2 groups were also similar in their clinical characteristics after 6 months, with no significant differences in glycemic control or in the use of CGM or insulin pumps (Table 2).

**Table 1:** Demographic characteristics of study participants at enrollment.

Characteristics		Values		
		Control (n=20)	Intervention (n=20)	*P* value^a^
Age (years), mean (SD)		8.4 (3.3)	11.3 (3.9)	.02
**Race, n (%)**				.31
	White	14 (70)	12 (60)	
	Non-White	4 (20)	2 (10)	
	Unknown	2 (10)	6 (30)	
**Hispanic ethnicity, n (%)**		2 (10)	4 (20)	.66
**Insurance, n (%)**				.87
	Public	10 (50)	9 (45)	
	Private	9 (45)	10 (50)	
	Self-pay	1 (5)	1 (5)	

^a^Calculated using the Fisher exact test or the Student *t* test.

**Table 2:** Clinical characteristics of study participants after 6 months.

Characteristics	Values		
	Control (n=20)	Intervention (n=20)	*P* value^a^
CGM^b^ use, n (%)	19 (95)	17 (85)	.60
Insulin pump use, n (%)	8 (40)	4 (20)	.30
HbA_1c_^c^, mean (SD)	8.3 (1.1)	8.2 (1.3)	.77

^a^Calculated using the Fisher exact test or the Student *t* test, as appropriate.

^b^CGM: continuous glucose monitor.

^c^HbA_1c_: hemoglobin A_1c_.

### Feasibility of Remote Monitoring

Remote glucose monitoring was successfully established for 100% of the families in the intervention group via Tidepool and for 85% of the families via Epic. Difficulties establishing remote monitoring via Epic resulted from problems creating full access MyChart accounts for 3 of the participants due to medical-center–specific policies, rather than technical errors. The issues encountered for these 3 participants were later resolved at a system level, but not during the intervention period for this study.

### Survey Findings

**Figure 2:**
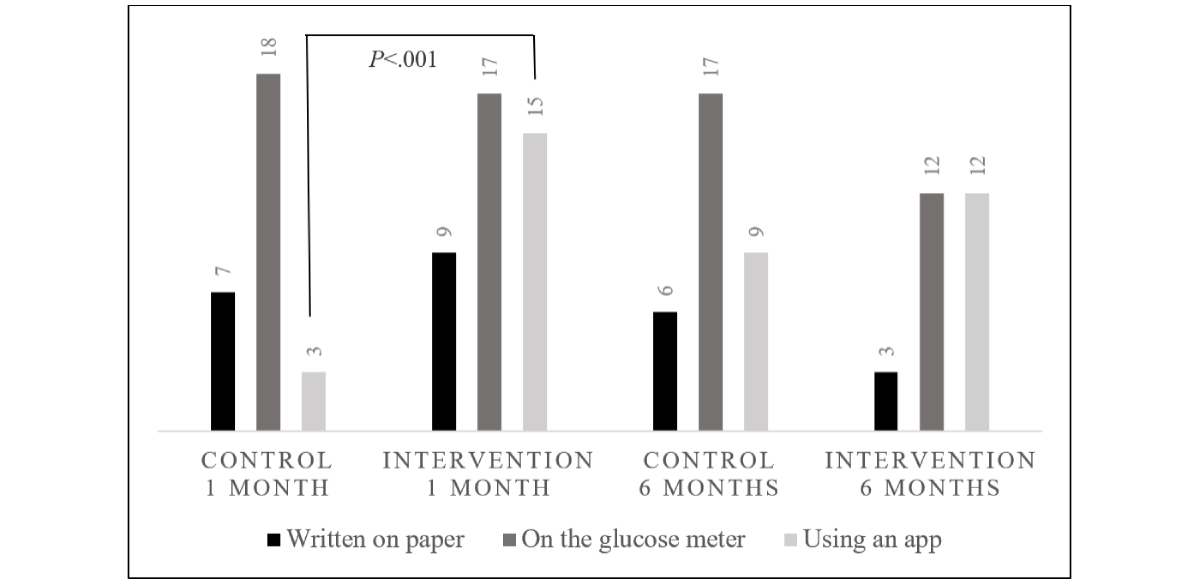
Survey responses to “How do you review your child’s glucose levels (select all that apply)?” All *P* values >.05, except as shown.

One-month surveys were completed for all participants, and 6-month surveys were completed for 39 of the 40 participants. At both 1 month and 6 months, all families in the intervention and control groups reported that they reviewed their glucose values at least daily. Their chosen method for reviewing these values differed significantly at 1 month but not at 6 months (Figure 2), with greater use of mobile apps in the intervention group as compared with the control group at 1 month after diagnosis. In Figure 2, survey responses to “How do you review your child’s glucose levels (select all that apply)?” were analyzed. The Fisher exact test was performed for each response, comparing control and intervention groups at each time interval, and comparing time intervals within each group.

**Figure 3:**
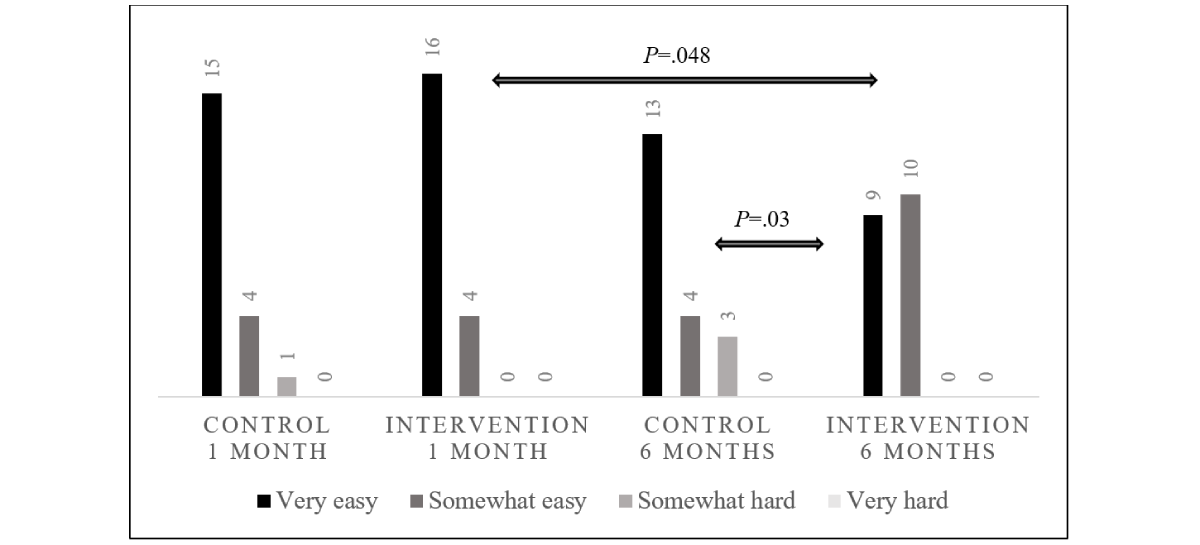
Survey responses to “How easy is it to discuss your child’s glucose levels with his/her diabetes team between clinic visits (select one)?” All *P* values >.05, except as shown.

At 1 month after diagnosis, the majority of participants in both groups felt it was “very easy” to contact the clinical diabetes team for help between visits. By 6 months, this perceived ease of contact had decreased significantly for families in the intervention group in comparison with their own responses at 1 month (during the intervention period) and compared with the control group responses (Figure 3). In Figure 3, the survey responses to “How easy is it to discuss your child’s glucose levels with his/her diabetes team between clinic visits (select one)?” were analyzed. The Fisher exact test was performed for the overall distribution of responses, comparing control and intervention groups at each time interval, and comparing time intervals within each group.

## Discussion

### Primary Findings

This pilot study demonstrates the feasibility of remote glucose monitoring among pediatric patients with newly diagnosed T1D using Bluetooth-capable glucose meters and Wi-Fi–enabled mobile devices. Remote glucose monitoring has been utilized previously for adults with type 2 diabetes [9,10], and for children with T1D utilizing insulin pumps [11] or CGM devices [12]. To our knowledge, this is the first published study to utilize remote glucose monitoring via finger-stick glucose meters among pediatric patients with newly diagnosed T1D.

Our survey results suggest that the use of remote monitoring accelerated patients’ and caregivers’ adoption of mobile health apps for the self-review of glucose data during the first month after diagnosis. The use of remote monitoring did not appear to alter the frequency of glucose self-review during the first 6 months after diagnosis, because all control and intervention families reported daily review of glucose data during this time; however, it is possible that differences might emerge with a longer duration of follow-up. Interestingly, ease of glucose review and insulin dosage adjustment via remote monitoring was such that after the completion of the intervention period, routine methods for contacting the diabetes care team were perceived as less easy by the intervention group, as demonstrated on their 6-month surveys.

In addition to these reported findings, remote glucose monitoring was also noted to improve the efficiency of provider workflows in several ways during the study. Easy access to remotely shared glucose data reduced the amount of time spent verbally recounting glucose values by telephone with families, enabling a greater amount of time to be spent on the discussion of glucose trends and diabetes management behaviors. In addition, the ability to review glucose data for all participants prior to contacting them improved provider efficiency by facilitating focus on those children most in need of insulin dose adjustments. Finally, the wide-scale adoption of video and telephone care necessitated by the COVID-19 pandemic—which began midway through this study—further highlighted the utility of remote monitoring from a provider standpoint by facilitating easy access to patient-generated data during telehealth appointments.

It is important to note that CGM technology is increasingly utilized in pediatric T1D and can be successfully initiated within several weeks of diagnosis [13]. However, we sought to provide remote monitoring starting at the time of hospital discharge (2-3 days after diagnosis) and continuing for 2-6 weeks afterward, during which time payors and suppliers have typically not yet authorized or provided CGM devices. We therefore evaluated the feasibility of remote monitoring of intermittent finger-stick glucose measurements. Because finger-stick glucose meters are universally available at the time of diagnosis, this study’s results have broad applicability for patients with newly diagnosed T1D. In addition, the outcomes we evaluated in terms of patient experience are likely generalizable to patients utilizing CGM devices, because they center on the patient’s interactions with mobile device apps and clinical providers rather than with the glucose monitoring device itself.

### Limitations

This study was designed as a pilot and feasibility study, and as such, it was limited in its size and scope. Because the assignment to the intervention was not randomized, the participants were able to self-select into intervention and control groups to a certain extent. Although the intervention participants were not required to own a mobile device or have knowledge to set up the necessary apps and data relays (since these were provided by research staff), children and families choosing to participate in the intervention may have had higher comfort using mobile devices and apps. The higher mean age of the participants in the intervention group may reflect this difference, as adolescent patients tend to have higher digital literacy than younger children. This type of self-selection reflects the realities of clinical practice, and this study therefore retains high validity in demonstrating the feasibility of remote glucose monitoring among willing participants. However, our results should not be generalized to infer what the experience of remote monitoring would be among all children with newly diagnosed T1D. In addition, the processes and outcomes for relaying glucose data into the EHR in this study may not be generalizable to practices utilizing non-Epic EHR systems, which may import and display glucose data differently. Furthermore, although our study population was diverse socioeconomically (50% publicly insured), 65% of the participants were of White racial backgrounds and 100% were English speaking, which limits the generalizability of our findings for minority demographic groups. Finally, it is important to note that this study’s survey questions were not specifically validated for use in this population and clinical context, and the survey findings should therefore be interpreted primarily as hypothesis-generating, to be used in developing future research studies.

### Future Directions

The purpose of any pilot study is to explore whether the intervention may be of benefit if applied on a larger scale. In the case of our remote monitoring intervention, translation from research into standard practice is feasible, but requires that several challenges be addressed.

On the patient side, Bluetooth-capable glucose meters are fortunately now approved by most if not all payors, and most brands have developed corresponding mobile apps that can collect glucose data and relay it to the cloud as well as to other compatible programs on a mobile device. However, the patient must have a personal mobile device with Bluetooth and internet capability, which can download and run multiple digital health apps simultaneously. Although it is preferable that this device remain with the patient the majority of the time (which can be problematic, particularly for school-aged children who may not have their own mobile phones), glucose data can be synced from the meter to the mobile device at a later time with full data retention. This is an advantage over some continuous glucose monitors that can only store up to 3 hours of data for later transmission to a mobile device. The data relay into the EHR in this study required the use of Apple Health and therefore could only be performed using an Apple device. As other health data apps such as Google Fit are expanding their compatibility, it is possible that this limitation will soon disappear. Battery life for the Bluetooth-capable meters and data use for the mobile devices proved to be an issue for some study participants, so in practice, patients would need to be warned about possible additional expenses for battery replacement and internet or cellular data transmission.

For providers, the primary challenge to broadening the use of this intervention is the need for new workflows and an expanded workforce. In most pediatric diabetes centers, the review of patient-generated glucose data is a reactive process for providers, performed in response to patient-initiated contact. The efficient use of remote monitoring could transform this to a provider-initiated process whereby data are reviewed on a regular basis and outreach to patients occurs based on predetermined criteria. Our study’s intervention did not significantly increase the provider workload for newly diagnosed patients (a cohort with whom frequent contact is routine); however, the expansion of remote monitoring to established patients with T1D would increase provider burden substantially by broadening the target population and by augmenting the frequency of contact with these patients. This type of expansion would necessitate a larger clinical workforce and the development of patient care algorithms which could be enacted primarily by nonphysician care team members. In addition, supplementary time and personnel would be needed to provide technical assistance and technology-related patient education—roles that were filled by research staff in this study—to assist with initiating remote glucose monitoring in clinical practice.

In addition to identifying specific patient and provider challenges to implementing remote glucose monitoring, this study generated several positive findings that deserve mentioning in the context of future directions. First, this study provided a proof-of-concept for importing glucose data directly into the EHR, but also demonstrated that the data relay was simpler to establish, and the visualization of data was superior via a diabetes-specific data platform (Tidepool), compared with the EHR. Therefore, although EHR integration of patient-generated data is an important goal for clinicians and health systems, future remote monitoring initiatives for diabetes will likely be most successful if they utilize the data visualization capabilities of existing third-party diabetes management tools and integrate these platforms with the EHR using single sign-on functionality. Second, our research team observed improved clinician efficiency using remote monitoring versus usual care. This observation should be explored quantitatively in future trials, comparing overall provider time alongside patient-centered outcomes to better understand the total benefits of remote monitoring protocols. Third, our study’s intervention had positive effects on the use of mobile diabetes apps and communication with diabetes providers after 1 month, but it is unclear if these benefits were due to the remote monitoring of diabetes data, the supply of mobile devices and orientation to diabetes apps, or the daily provider-initiated contact to families. Future, larger trials of remote monitoring would benefit from enrolling multiple intervention arms in order to separate the effects of these factors.

### Conclusions

This pilot study demonstrates the feasibility of remote glucose monitoring among pediatric patients with newly diagnosed T1D using Bluetooth-capable glucose meters and internet-connected mobile devices. The future application of remote monitoring to broader T1D populations hinges on the ability to establish passive, continuous data sharing via a broad array of mobile devices and glucose meters, and the development of provider algorithms for managing T1D populations on a continuous basis.

## Abbreviations

CGM: continuous glucose monitoring
EHR: electronic health record
PGHD: patient-generated health data
SMBG: self-monitoring of blood glucose
T1D: type 1 diabetes
UCD: University of California, Davis
